# Relationships between body condition score and ultrasound skin-associated subcutaneous fat depth in equine

**DOI:** 10.1186/1751-0147-57-S1-P12

**Published:** 2015-09-25

**Authors:** Severiano R Silva, Rita Payan-Carreira, Miguel Quaresma, Cristina M Guedes, Ana Sofia Santos

**Affiliations:** 1 Animal and Veterinary Research Center, CECAV, Universidade de Trás-os-Montes e Alto Douro, Vila Real, Portugal; 2 Escola Universitária Vasco da Gama, Coimbra, Portugal

## Introduction

Body composition is a fundamental management tool in equids (horse and donkey). Scoring body condition (BCS) and measurement of fat or tissue depths by ultrasound are the most commonly used methods to quantify and monitor body composition. The latter uses images which require interpretation and sometimes the boundaries between tissues are not obvious. In several studies, particularly for animals with thin subcutaneous fat (SF) deposits, the skin is included in the measures to reduce measurement errors.

## Objective

The objective of this work was to have a comprehensive relationship between SF plus skin (SF_skin) ultrasound measurement and the BCS from horses and donkeys.

## Material and methods

This study enrolled 43 animals (donkeys:16 and horses: 27). BCS was measured using a 9 point scale. Ultrasound images were obtained with an Aloka SSD 500V real time scanner and a linear 7.5 MHz probe, placed over the 13th thoracic vertebra. Captured images were analysed in ImageJ to obtain SF_skin measurement. Simple and polynomial regressions between BCS and SF_skin were calculated. Equations were evaluated with the determination coefficient (R2) and the root mean square error (RMSE).

## Results

Irrespective of species, the best fit was achieved with the polynomial model (R2=0.677, RMSE = 1.32). When the species was considered separately, the best fit was obtained by the polynomial model (R2=0.921; RMSE=0.648 and R2=0.772; RMSE=1.06, for donkeys and horses, respectively).

## Conclusion

The curvilinear nature of the relationship between BCS and SF_skin measurements limits the accuracy of BCS with increasing adiposity, which constrains the usefulness of BCS for a wider application.

